# Supplementary material for: Quantification and visualization of US racial geography using the National Racial Geography Dataset 2020
Source: PLoS One. 2024 Jul 25;19(7):e0307745. doi: 10.1371/journal.pone.0307745 (PMC11271910; doi:10.1371/journal.pone.0307745)
Supplement: Attachment — Submitted filename: Response to Reviewers.pdf [file pone.0307745.s002.pdf]

Considering the comments of both reviewers, we significantly rewrote the paper, including the changes to the title. We also wrote a new Discussion section of the paper that focuses on descibing the differences between analyzing and visualizing segregation and diversity using traditional approaches and our dataset with the RL methodology. We hope this new version clearly states the novelty of our approach compared to the traditionally used methods.

Editor comments

In addition, I find your statement “the existing literature primarily focuses on quantifying residential diversity and segregation within large metropolitan areas” is true only for public data. The restricted use of census data contain geographic information down to the block level for each individual so theoretically racial diversity can be calculated precisely at the block level using the confidential census data.

As the editor mentions, the individual-level data are confidential and have restricted access. Thus, it cannot be commonly used in racial diversity and segregation analysis. The lack of individual-level data prompts the researcher to develop methods that can be applied to the census aggregated data. The existing literature focuses on analyzing segregation and diversity using census-aggregated data that are openly available.

Reviewer 1

Reviewer #1: This research aims to present a fine-grained dataset of racial demography for the U.S. in 2020. Due to privacy concerns, the existing studies generally measure racial segregation at the level of census tract, which is less accurate. Based on their previous research on racial landscapes, the authors transform vector-based census data into high-resolution grid data. This dataset potentially promotes the development of racial demography. However, there are several major issues needed to be addressed.

We would like to make several points here.

(1) The grid used in the present paper is *monoracial*, meaning each cell groups inhabitants of a single race only. This is in contrast to previous grids that are all multiracial.

(2) The high resolution of the grid has been a distinct feature of previous multiracial grids; here, the high resolution is a secondary feature, and monoraciallity is the primary feature.

(3) This paper does not introduce a monoracial grid (it has already been introduced). Instead, it introduces the NRGD dataset that allows researchers to calculate the segregation of an arbitrary region within the conterminous US without the technical knowledge needed to generate a monoracial grid. Of course, the present paper contains a brief on the RL method (a method of generating a monoracial grid and using it for the zoneless calculation of segregation). Still, it is a non-technical brief that focuses on key concepts.

1. Current metrics for racial diversity and segregation should be examined. The authors need to engage with the extant literature concerning proxies of racial demography, compare different metrics and justify the choice of metrics of this research.

The novelty here is not a better metric of segregation but the ability to calculate segregation for an arbitrarily defined region of interest (ROI) not restricted to a census aggregated area divided into smaller census aggregated areas – a restriction of the traditional method. The need for such functionality is self-evident, but we still justify it in several places in the revised paper. To have such functionality, the segregation metric used in the paper has to adhere to the transformed data in order to work due to the need for a "new" metric. Thus, a review of existing segregation metrics would be out of topic in this paper as they do not apply to the monoracial grid. The revised paper also states that using a set of 50 US metropolitan areas, the rank correlation between the metric *NMI* used to assess segregation from a pattern and the index *H* used in traditional calculation assessment is 0.92. Thus, *NMI* measures segregation in the same sense as *H* but is not restricted to census aggregated areas.

2. Accuracy of this dataset should be tested carefully. What is the accuracy of this dataset? Does the accuracy vary spatially? If so, why? Due to the stochastic nature of this approach, how does the number of realization affect the accuracy of the dataset? Does the dataset have any accuracy gain compared to other datasets/approaches?

The question needs to be more specific. The term "accuracy of the dataset" is too broad as the dataset consists of many layers representing different variables. The accuracy of transformed data, the monoracial grid, is, by design, the same as the accuracy of census block data; integrating all cells in the block yields the racial composition as listed by Census block-level data. In other words, a sub-census spatial distribution is arbitrary, but this is OK, as our goal was not to increase spatial accuracy below the census block but to change the structure of the data so the

segregation metric can be calculated from a pattern. This is stated in the revised paper.

The accuracy of the segregation metric is also limited by the accuracy of census block data. See our reply to the next question on the issue of how a stochastic approach influences the accuracy of the segregation metric.

Due to the stochastic nature of this approach, how does the number of realization affect the accuracy of the dataset?

As in all stochastic approaches, an ensemble mean is more accurate (the standard deviation is smaller) for a larger number of realizations. This issue has been discussed in the technical paper on the RL methodology. (Dmowska et al. 2020, https://www.sciencedirect.com/science/article/abs/pii/S0143622819310367). The result is that accuracy is more sensitive to the size of the area over which the value of the segregation metric is computed than the number of realizations. We determined that with 50 realizations, the accuracy of the metric for areas larger than a census block is very high.

To illustrate to the reviewer that the number of realizations does not affect the results, we chose 50 counties and, for each county, calculated 50 realizations. In the figure below, the black points present the segregation metrics calculated for each realization; the red point is a mean calculated from 50 realizations. It can be observed that the deviation of metrics values is indeed small regardless of segregation metrics values.

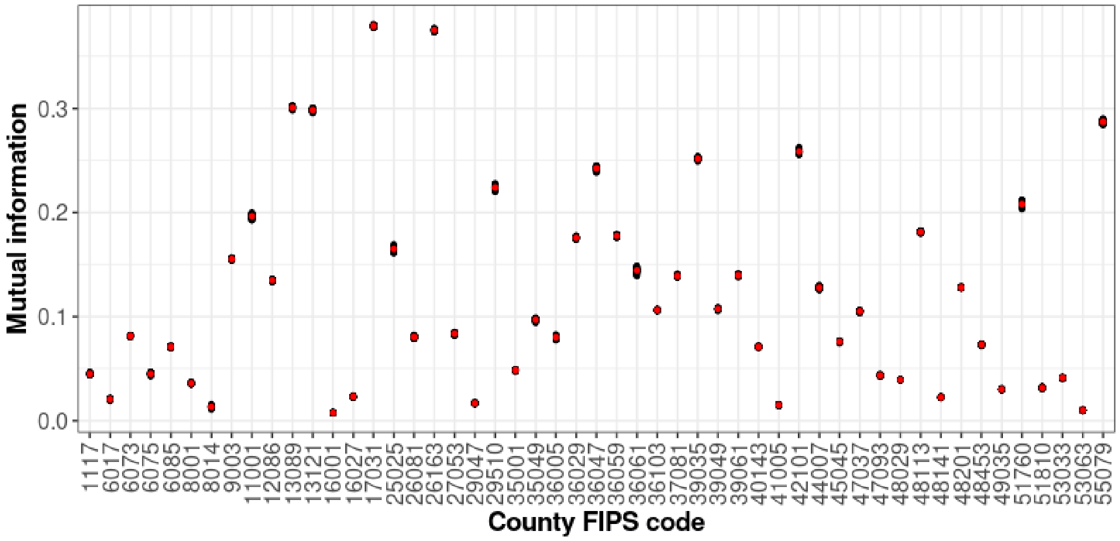

Some minor points:

3. Three metrics (NH, MI, and NMI) should be compared in a table. What are their strengths and weaknesses? What is the relationship between each metric and segregation/diversity (i.e., racial spatial pattern)? Are they mutually reinforcing?

In the revised version of the paper, we include a separate subsection in the Material and Methods that describes and explains those 3 metrics. They are not measuring the same property, so their comparison in a table would not provide any more information. N_H measures diversity; it is just an entropy expressed in an easier-to-interpret manner. *MI* measures a degree of clumping of the same-race cells in a pattern; clumping of racial pattern corresponds to racial segregation. By its design, *MI* increases with the increase in the number of different racial sub-populations. Thus, according to *MI*, a completely segregated city with three significant races is more segregated than a completely segregated city with two races. *NMI* is the *MI* relative to the diversity of the region measured by the entropy (NMI=MI/E = MI/(Log N_H)). Thus, according to $_{NMI}$, a completely segregated city with three significant races has the same level of segregation as a completely segregated city with two races. This is explained in the revised paper.

4. There should be more discussion of the limitations of the dataset.

The limitations of the RL method and the limitation of the NRGD dataset are two different issues. Both RL and NRGD are introduced to remove limitations of the traditional method and census aggregated data, respectively; therefore, by design, they have fewer limitations than the traditional approach.

Keeping in mind that the primary quantification goal of RL is to enable assessing segregation for an arbitrary region, the only limitation (stated in the paper) is that the size of the region needs to be larger than a census block. The limitation of the dataset is self-explanatory from the list in Table 1. The pre-calculated grids for local diversity and segregation (rows 2 and 3 in the revised Table 1) are calculated only for square areas at 10 different scales. In reality, this is not a significant limitation. To calculate segregation and diversity of arbitrary area, which is not pre-calculated, a user has to choose an ROI from race and density grids (rows 4 and 5 in the revised table 1) and do calculations in the R package raceland. Is it a limitation? We don't think so because the package is freely available and relatively easy to use.

REVIEWER 2

Reviewer #2: The aim of this study was to improve the accessibility and effectiveness of 435 racial geography studies within the GIS environment. The authors sought to convert vector-based census data into high-resolution raster data and present a fine-grained dataset of racial demographics for the United States in 2020. This is a very interesting and important topic to discuss, but I do not think the argument is entirely new. Again, this is probably not entirely new and can certainly be found in the existing literature, but it is worth collecting in one place along with the above point. While I recognize the roles of this paper, I am not sure its contribution is original enough for the PLOS ONE standard.

The work presented in this paper is entirely new. Although descriptions of quantitative methods of assessing racial segregation abound in the literature, they all restricted to census-aggregated areas divided into other census-aggregated areas. Thus, a traditional method can assess the segregation of a county or a metropolitan statistical area but not, for example, a zip code or a congressional district.

Only recently was the Racial Landscape (RL) method published that allows for assessing segregation of an area of arbitrary size and shape. The RL is unlike any previous methods as it uses a stochastic approach to transform census block racial data into sub-block cells of monoracial cells, thus converting racial data to a spatial pattern from which a zoneless assessment of segregation is possible. This stochastic underpinning of RL, unfamiliar to the demographic community, presents a barrier to the adaption of RL in racial studies, thus missing the opportunity to perform studies not possible under the traditional framework. The present paper aims to introduce the NRGD dataset, which contains multiple layers of information pre-calculated using the RL for the entire conterminous US in 2020. This makes it possible to take advantage of the RL without necessarily a technical understanding of its stochastic underpinning.

1, Theoretically, the authors argue that recent studies expose racial geography in the emergent context of the U.S. as a new apparatus of regionalism, However, this dichotomy is not convincing, as regionalism and the role of the market and the state are intensely linked and debated in the literature.

We don’t understand this remark, but certainly, it has no relation to the core of our paper. Likely, the reviewer refers to the introduction, where we describe some regional differences in the distribution of different races. This was done only to point

out that the spatial distribution of different racial populations is not simple. This description is no longer present in the revised manuscript to avoid confusion.

2, As for the literature review, perhaps more attention should be paid to this in the introduction, as I found the existing literature strangely under-cited. Particularly in the literature review, these types of sources, the racial demography and regionalism literature are cited more extensively.

As we have already explained in our answer to the first question, the methodology we work with has no technical connection to methodologies described in past literature. Thus, a review of past literature would be out of place. Furthermore, the RL method itself is not a focus of this paper (although we give a non-technical brief on the RL method). It was a focus of an earlier paper (Dmowska et al. 2020, https://www.sciencedirect.com/science/article/abs/pii/S0143622819310367) where several citations were given in which standards methods of quantification of racial segregation are reviewed. In this paper, we focus on the construction of the NRGD dataset and the demonstration of its usefulness for tasks for which the standard methodology is not adequate.

3, Data: The accuracy of the data set should be justified, how do the authors intend to convince their peers of this data? The reasons for choosing this data set should be carefully explained.

The reason for choosing racial data represented as a monoracial grid is clearly explained in the paper (especially in the revised manuscript) and has nothing to do with accuracy. The reason is that a monoracial grid (a spatial pattern) is the form of racial data supporting zoneless segregation calculation, while previously used aggregated multiracial format does not. The two use cases presented in the paper clearly illustrate the advantage of the ability to calculate segregation for arbitrary areas, including square blocks.

As for the actual accuracy of monoracial gridded data, by design, it is the same as the accuracy of census block data. Integrating all cells in the block yields the racial composition as listed by census block-level data. Redistribution of block inhabitants to sub-block cells is not to increase the accuracy but to form a pattern.

4, Method: The methods used to convert vector-based census data into high-resolution raster data in this study should be explained and discussed, what are the advantages of the method chosen in this study?

As we already explained, a technical description of RL construction is not the topic of this paper. The full description is available in the previous paper (Dmowska et al. 2020, https://www.sciencedirect.com/science/article/abs/pii/S0143622819310367).

We have already explained that the ability to calculate the segregation of an arbitrary area without using any zones is the key advantage of the method. The additional advantage is the tight integration of visualization and quantification of racial geography.

4, Discussion and conclusion: the conclusion should address the research questions and deal with the current literature, which are the basic points of the research paper. The discussion section should also discuss the limitations of this research and its methods.

This is a kind of paper that does not have a specific research question. Such papers are explicitly allowed by PlosOne. The purpose of the paper is to introduce an extensive dataset that would expedite and improve studies in which the calculation of racial segregation anywhere in the US and on any scale is needed.

In the revised paper, we rewrote the Discussion section. It now focuses on describing the comparison between standard methods and our approach.

5, the English language should be checked more carefully as there are numerous English grammatical and typographical errors in the paper.

We check the English in the revised version.
