# Supplementary material for: Quantification and visualization of US racial geography using the National Racial Geography Dataset 2020
Source: PLoS One. 2024 Jul 25;19(7):e0307745. doi: 10.1371/journal.pone.0307745 (PMC11271910; doi:10.1371/journal.pone.0307745)
Supplement: Attachment — Submitted filename: Response_to_reviewer.pdf [file pone.0307745.s003.pdf]

Response to reviewer

Reviewer #1: The authors largely addressed my previous concerns. However, there is one remaining issue. In response to my query regarding the impact of the stochastic nature of their approach on the accuracy of the dataset, the authors demonstrated that the number of realizations barely affects the mutual information (i.e., the segregation metrics). Despite this clarification, the effect of the number of realizations on diversity metrics remains unexplored and warrants further investigation. Although this topic was briefly mentioned in section 2.4 of their paper published in Applied Geography (https://www.sciencedirect.com/science/article/abs/pii/S0143622819310367), the discussion lacked comprehensive detail. Therefore, a more thorough analysis of the stochastic characteristics of RL and its implications for the dataset should be incorporated into this manuscript. This inclusion will not only enhance the completeness of the research but also provide a clearer understanding of how the number of realizations influences the broader outcomes of the study.

As it is mentioned by the reviewer, we clarify that the number of realizations does not affect the segregation metrics. The same is true about the racial diversity metric. We perform test using 51 counties characterized by different spatio-racial pattern, and the results of this test are available in section Racial Landscapes at https://socscape.edu.pl In the revised manuscript we added the lines 92-106 to clarify this issue, and for further explanation we included also link to the https://socscape.edu.pl/index.php?id=racial-landscapes

The conclusions from our test are as follows:

- In our sample of 51 counties the mean segregation metric MI calculated using 50 realizations change from 0.007 to 0.379 (with the standard deviation between 0.0002-0.002), and the mean diversity metric E range from 0.91 to 2.13 (with the standard deviation between 0.0003 - 0.005).
- In Figure 1 (please see the document at https://socscape.edu.pl/index.php?id=racial-landscapes) the black points present the segregation metrics calculated for each of realization; the red point is a mean calculated from 50 realizations. The difference between mutual information values (black point) is so small that is comparable to the symbol size in this plot (symbol size is 0.5 mm), for some counties the black points are covered by the red point (mean value). The conclusions from this plot is that the deviation of segregation metrics values is small regardless of segregation metrics values.
- In Figure 2 (please see the document at https://socscape.edu.pl/index.php?id=racial-landscapes) the black points present the diversity metrics calculated for each realization; the red point is a mean calculated from 50 realizations. It can be observed that the deviation of metrics values are even smaller than for the segregation metric MI.
- The Spearman correlation between segregation metric calculated using NRGD (1 realization), and the mean values from 5, 10, 20, 30, 40, and 50 realizations range from 0,9997 to 0,9999. It indicates that the rankings based on segregation metrics are almost identical. The further investigation shows that 2 pairs of counties switch their position.
- For example, comparing the rankings based on segregation metrics calculated using 50 realizations, and 1 realization derived from NRGD:
  - counties Shelby, AL and San Francisco, CA switch the positions between 15 and 16. (Shelby, AL: *MI* based on 50 realizations is equal to 0.44835, and based on NRGD is 0.04534; San Francisco, CA: *MI* based on 50 realizations is equal to 0.04490, and based on NRGD is 0.04512)
  - counties Bronx, NY and Kent, MI switch their positions between 22 and 23. (Kent, MI: *MI* based on 50 realizations is equal to 0.08036, and based on NRGD is 0.0796; Bronx, NY: *MI* based on 50 realizations is equal to 0.08013, and based on NRGD is 0.08039)
- The Spearman correlation between diversity metric calculated using NRGD, and the mean values from 5, 10, 20, 30, 40, and 50 realizations range from 0,9996 to 0,9997.

- For example, comparing the rankings based on diversity metrics calculated using 50 realizations, and 1 realization derived from NRGD:
    - counties Bronx, NY and Bermallillo, NM switch the positions between 25 and 26. (Bronx, NY: *E* based on 50 realizations is equal to 1.6878, and based on NRGD is 1.6838; Bermallillo, NM: *E* based on 50 realizations is equal to 1.6849, and based on NRGD is 1.6843)
    - counties Santa Clara, CA and San Diego, CA switch the positions between 33 and 34. (Santa Clara, CA : *E* based on 50 realizations is equal to 1.8795, and based on NRGD is 1.8776; San Diego: *E* based on 50 realizations is equal to 1.8791, and based on NRGD is 1.8784)
