# Supplementary material for: Quantification and visualization of US racial geography using the National Racial Geography Dataset 2020
Source: PLoS One. 2024 Jul 25;19(7):e0307745. doi: 10.1371/journal.pone.0307745 (PMC11271910; doi:10.1371/journal.pone.0307745)
Supplement: Attachment — Submitted filename: review report for PLOS ONE.docx [file pone.0307745.s001.docx]

The aim of this study was to improve the accessibility and effectiveness of 435 racial geography studies within the GIS environment. The authors sought to convert vector-based census data into high-resolution raster data and present a fine-grained dataset of racial demographics for the United States in 2020. This is a very interesting and important topic to discuss, but I do not think the argument is entirely new. Again, this is probably not entirely new and can certainly be found in the existing literature, but it is worth collecting in one place along with the above point. While I recognize the roles of this paper, I am not sure its contribution is original enough for the PLOS ONE standard.

1, Theoretically, the authors argue that recent studies expose racial geography in the emergent context of the U.S. as a new apparatus of regionalism, However, this dichotomy is not convincing, as regionalism and the role of the market and the state are intensely linked and debated in the literature.

2, As for the literature review, perhaps more attention should be paid to this in the introduction, as I found the existing literature strangely under-cited. Particularly in the literature review, these types of sources, the racial demography and regionalism literature are cited more extensively.

3, Data: The accuracy of the data set should be justified, how do the authors intend to convince their peers of this data? The reasons for choosing this data set should be carefully explained.

4, Method: The methods used to convert vector-based census data into high-resolution raster data in this study should be explained and discussed, what are the advantages of the method chosen in this study?

4, Discussion and conclusion: the conclusion should address the research questions and deal with the current literature, which are the basic points of the research paper. The discussion section should also discuss the limitations of this research and its methods.

5, the English language should be checked more carefully as there are numerous English grammatical and typographical errors in the paper.
